# A Photophysical Deactivation Channel of Laser-Excited TATB Based on Semiclassical Dynamics Simulation and TD-DFT Calculation

**DOI:** 10.3390/molecules23071593

**Published:** 2018-06-30

**Authors:** Wenying Zhang, Jian Sang, Jie Cheng, Siyu Ge, Shuai Yuan, Glenn V. Lo, Yusheng Dou

**Affiliations:** 1 Chongqing Key Laboratory of Big Data for Bio Intelligence, Chongqing University of Posts and Telecommunications, Chongqing 40065, China; zhangwenying@cqupt.edu.cn (W.Z.); 2013212035@stu.cqupt.edu.cn (J.S.); 2014212214@stu.cqupt.edu.cn (J.C.); gesiyu96@163.com (S.G.); 2 Department of Physical Sciences, Nicholls State University, P.O. Box 2022, Thibodaux 70310, LA, USA; glenn.lo@nicholls.edu

**Keywords:** semiclassical dynamic, TD-DFT, nonradiative deactivation, vibrational relaxation, charge transfer, energetic materials, TATB

## Abstract

A deactivation channel for laser-excited 1,3,5-triamino-2,4,6-trinitrobenzene (TATB) was studied by semiclassical dynamics. Results indicate that the excited state resulting from an electronic transition from the highest occupied molecular orbital (HOMO) to the lowest unoccupied molecular mrbital (LUMO) is deactivated via pyramidalization of the activated N atom in a nitro group, with a lifetime of 2.4 ps. An approximately 0.5-electron transfer from the aromatic ring to the activated nitro group led to a significant increase of the C–NO_2_ bond length, which suggests that C–NO_2_ bond breaking could be a trigger for an explosive reaction. The time-dependent density functional theory (TD-DFT) method was used to calculate the energies of the ground and S_1_ excited states for each configuration in the simulated trajectory. The S_1_←S_0_ energy gap at the instance of non-adiabatic decay was found to be 0.096 eV, suggesting that the decay geometry is close to the conical intersection.

## 1. Introduction

Energetic materials refer to organic substances that can store a large amount of chemical energy. Under the action of external stimuli, such as shockwave and light, energetic materials readily decompose and release energy [1,2,3]. The energy release mechanism is very complicated. In general, irradiation by an ultraviolet or visible laser can be used to electronically excite a molecule, which can then undergo a series of photochemical and photophysical processes, including radiative transitions and nonradiative deactivation. Nonradiative deactivation can involve internal conversion and intersystem crossing; internal conversion is considered to be a crucial first step for an explosive decomposition reaction because the vibrational energy generated by the deactivation can lead to the breaking of chemical bonds, triggering a chain reaction that eventually releases the stored chemical energy. Nitro-explosives are among of the most important energetic materials. The incomplete combustion of nitro-explosives can lead to the release of aromatic nitro compounds into the environment, which may lead to biological mutation and carcinogenesis. Photochemical degradation is the most common natural elimination method [3]. Thus, a study of the decay properties of excited states of nitro compounds has important civil and military applications.

The presence of nitro groups significantly changes the photochemical and photophysical behavior of molecules and affect the stability and sensitivity of energetic molecules [4,5,6]. Recent literature shows [7,8,9,10,11,12,13,14,15,16,17,18] excited energetic molecules deactivated from the S_1_ excited state to the S_0_ ground state through a conical intersection (CI). Experimental and theoretical studies by Bernstein and coworkers [6] found that the conical intersection plays an important role in the non-adiabatic decay of the energetic molecule dimethylnitramine (DMNA). They used the complete active space self-consistent field (CASSCF) method to optimize the (S_1_/S_0_) _CI_ point structure of the DMNA molecule along the nitro–nitrite isomerization reaction coordinates. This isomerization process was predicted to take place through a “loose transition state-like geometry”. Bernstein’s group studied the non-adiabatic transitions of many energetic molecules [8,9,10,11,12,13,14] and found that their (S_1_/S_0_) _CI_ are similar in structure. Soto, Arenas and collaborators [15,16,17] used the complete active space with second-order perturbation theory (CASPT2) to predict NO_2_–ONO isomerization in nitramide molecules through a (S_1_/S_0_) _CI_; a similar CI structure in the nitromethane molecule has led to the nitro elimination reaction. The aforementioned theoretical studies were static quantum calculations based on single-point optimization or potential energy surface scanning according to a simple reaction coordinate; time-resolved dynamics simulations were not reported. So far, only Ghosh et al. [17] used ab initio dynamics simulations to study small molecule energetic materials, such as nitromethane, DMNA, and methylnitrate; they found the S_1_ excited states of all the energetic molecules decayed to ground state through an ultrafast internal conversion with a lifetime shorter than 500 fs.

1,3,5-Triamino-2,4,6-trinitrobenzene (TATB) is a photosensitized insensitive explosive that is widely used in defense, mining, construction, and other fields. Photochromism, which is attributed to the photodecomposition of TATB, is observed under light irradiation [19,20,21,22,23,24]. The excited state of the TATB molecule is regarded as the important initial step of photodecomposition. Time-dependent density functional theory (TD-DFT) was applied to optimize the structure of S_1_ and T_1_ in TATB by Xiong et al. [23]. It was proposed that intersystem crossing from S_1_ to T_1_ is followed by a NO_2_−ONO isomerization. Chu et al. [23] utilized transient absorption spectroscopy and TD-DFT calculation to investigate the dynamics properties of excited states of TATB. They found structural changes, charge transfer properties, and the lifetimes of the excited states; their work laid a foundation for the study of the TATB excited states. However, the TD-DFT method, which is based on a single reference state, cannot correctly describe the structure of the non-adiabatic CI and the chemical bond breaking. It is difficult to build a complete potential energy surface and obtain information pertaining to the photochemical deactivation mechanism. The CASSCF/CASPT2 method has been widely used to study the state–state interaction and has achieved good precision in single-point optimization and excitation energy calculations. However, the active space contains a limited number of electrons and orbitals and can only be used to study excited states of small energetic molecules [25,26,27,28], from which the properties of larger energetic molecules are inferred. It is very hard to directly deal with larger aromatic systems such as TATB molecules. Even if some electrons and orbitals can be neglected to reduce the active space, it is difficult to select which ones to neglect, because the molecular structure of the non-adiabatic coupling point on the potential energy surface is unknown. Ab initio excited state dynamic studies of large molecules incur tremendously high calculation costs.

Correct understanding of the mechanism of a chemical reaction requires clarification of how each atom moves during the conversion from reactant to product. However, for a molecule with as many atoms as TATB, it is difficult to take into account the overall movements with CASSCF and TD-DFT. The ab initio dynamics methods, which were recently reviewed by Shalashilin and coworkers [29], are good candidates for addressing this problem, since a large number of degrees of freedom could be included. In this study, the semiclassical electronic radiation ion dynamics (SERID) [30,31] model was used to simulate the decay process of TATB excited states and track the movement of each atom, as well as the energy change over time. By comparing the findings with experimental and other theoretical results, we were able to identify a deactivation channel for the TATB S_1_ state.

## 2. Results and Discussion

To obtain an initial geometry of the TATB molecule for dynamics simulation, Gaussian 09 [32] was run using MP2/6-311+g(2d,2p) to generate an optimized geometry. Then, a 1000 fs dynamics run was performed in SERID for this single molecule with constant temperature and pressure. Structures at 10 equal time intervals were saved. From these, 10 trajectories can be simulated by taking each of the structures as the initial geometry, coupled with a laser pulse, for a SERID run. The average orbital energies were found to be −6.4 eV, −6.4 eV, −3.2 eV, and −2.8 eV for HOMO-1, HOMO, LUMO, and LUMO+1, respectively; HOMO-1 and HOMO are degenerate orbitals because of molecular symmetry; this is also observed in the calculation at the b3lyp/6-311++g(d,p) level [24]. Femtosecond laser pulses were applied to the equilibrium structure with a full-width at half maxima (FWHM) of 50 fs and photon energy of 3.2 eV (387 nm), which corresponds to the LUMO–HOMO energy gap. The excitation wavelength in the transient spectroscopy experiment of Chu et al. [23] was 400 nm (3.1 eV). The time step was set to 0.05 fs, and data were printed every 20 steps. To ensure sufficient relaxation time, we set our simulation time to 10 ps.

Out of 201 reaction trajectories, 37 trajectories stayed in ground state due to insufficient fluence, 26 trajectories stayed in excited electronic state (with vibrational relaxation), and 136 trajectories led to molecular fragmentations (such as –NO2, –O, and other carbonitride fragments, etc.) because of very high fluence. In these cases, excited state decay to the S_0_ state is not observed. The photophysical electronic decays S_1_←S_0_ were observed in two trajectories. Most of the trajectories (136/201) lead to molecular fragmentations, suggesting that TATB detonation is a very probable outcome of laser excitation. Detailed description of the photochemical reaction, i.e., photodissociation of TATB, will be described in another paper; this paper focuses on the non-adiabatic deactivation pathway of excited state. One trajectory with a laser frequency of 3.2 eV and fluence of 32.94 J·m^−2^ is reported here. In this trajectory, a photophysical deactivation channel of the TATB molecule via nitro relaxation is observed.

**Figure 1:**
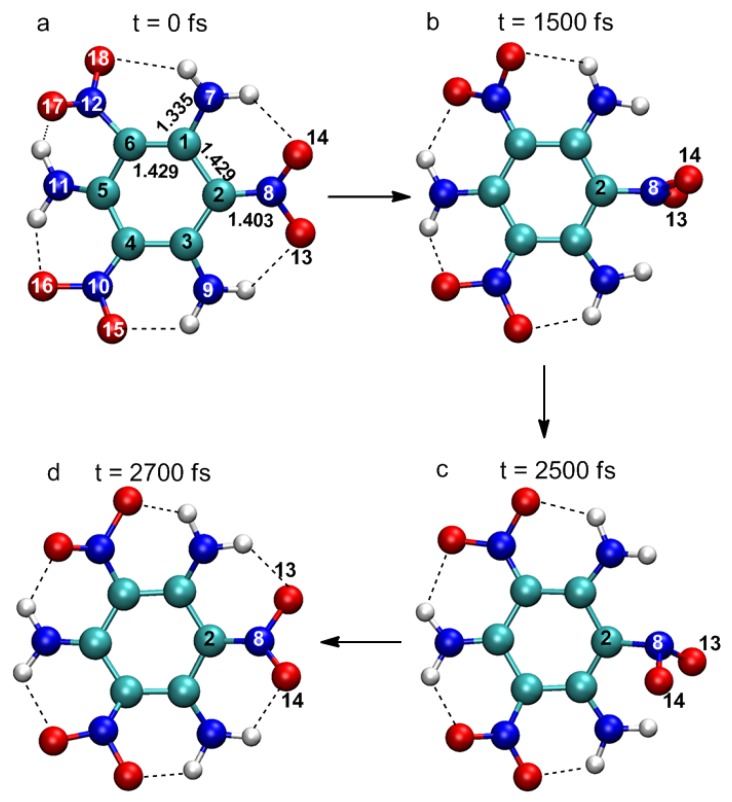
Snapshots for the deactivation of the excited state of the 1,3,5-triamino-2,4,6-trinitrobenzene (TATB) molecule following irradiation with a laser pulse of 3.2 eV photon energy and fluence of 32.94 J·m^−2^. Cyan: C, blue: N, red: O, white: H.

In Figure 1, four representative snapshots show important changes in molecular structure at different times in the simulation. Atomic labels are also shown in Figure 1a. At 0 fs, TATB molecules are basically planar structures, and there are a large number of intramolecular hydrogen bonds between adjacent –NO_2_ and –NH_2_ groups, which is the reason for the high melting point and good stability of TATB. Several key structural parameters are also identified in Figure 1a. The excited state is formed after the laser excitation. The main structural change is that the nitro group linked by the C_2_ atom rotates about the C_2_–N_8_ bond axis. The plane of O_13_–N_8_–O_14_ is nearly perpendicular to the plane of the benzene ring at 500 fs, while the displacements of other atoms are negligible. This suggests vibrational activation of the nitro group (hereafter referred to as the “activated nitro group” or ANG). At 2500 fs, the vibrational energy of ANG reaches a maximum, and a pyramidal structure is formed with N_8_ at the apex. At this time, the molecule decays to the ground state and the ANG rotates around the C_2_–N_8_ bond axis as the molecule returns to its original configuration. Significant structural changes were observed only in the ANG, indicating that the excitation is localized on the ANG. CASSCF level calculations of energetic molecules, such as nitrobenzene (NB) [25,26] and dimethylnitramine (DMNA) [27], show that excitation to the lowest excited states (n→π*) is localized at the nitro group; TD-DFT studies of TATB molecules [23] also yielded similar results. The ANG is not necessarily the nitro linked to C_2_ atom, it could also be another nitro linked to C_4_ or C_6_ atoms in the TATB molecule. For instance, the ANG was the nitro linked to the C_6_ atom in the other photophysical decay trajectory we obtained. 

**Figure 2:**
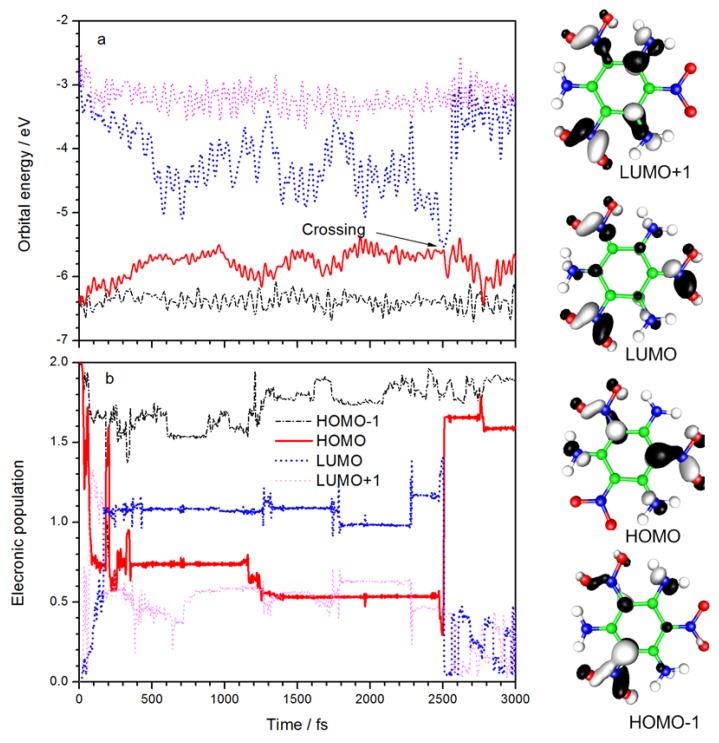
Variations with time of (**a**) the orbital energy and (**b**) electron populations of the HOMO-1, HOMO, LUMO, and LUMO+1 of TATB molecule.

The time evolution of the orbital energies and electronic populations of the HOMO-1, HOMO, LUMO, and LUMO+1 are summarized in Figure 2. Diagrams of the four frontier orbitals are also listed. The electron density in HOMO-1 is mainly distributed between C_4_ and the linked nitro group and is mainly distributed among C_2_, C_6_, and their linked nitro groups in HOMO. Both orbitals exhibit non-bonding (n) character. The electron in LUMO, which shows π* anti-bonding character, is distributed equally among the three NO_2_ groups, whereas the electron in LUMO-1 is distributed primarily between NO_2_ at the C_4_ and C_6_ positions and their corresponding NH_2_ groups. The lowest excited state should result from an n→π* excitation, similar to the smaller nitro-containing energetic molecules [25,26,27,28]. It can be seen from Figure 2a that the LUMO energy decreased rapidly after laser irradiation, and HOMO energy increased significantly at the same time. It means the electronic transition from HOMO to LUMO is the dominant excitation. After 500 fs, the energy of HOMO and LUMO fluctuated between −4.4 and −5.8 eV, respectively. At 2500 fs, the energy gap of LUMO–HOMO reaches a minimum of 0.04 eV, and the orbital energies rapidly return to their original values. Figure 2b shows the variation of frontier orbital electron population with time. After laser irradiation, about one electron is excited from HOMO to LUMO, and a small amount goes to the LUMO+1. The excited state decays to ground state after the “crossing” point, as marked in Figure 2a, which corresponds to a LUMO-to-HOMO transition in Figure 2b. The laser frequency used in simulation (3.2 eV) is close to the excitation wavelength used in the experiment (400 nm, i.e., 3.1 eV), suggesting that the density-functional-based tight binding (DFTB) method with parametric quantification is adequate for the calculation of electron energy. Due to a limitation of the Ehrenfest dynamics theory, the excited states decay to a mixed state rather than a pure state, so there is a small amount of electronic population of other orbitals. The simulation shows that with a 100 fs laser duration, the excited state deactivation takes place at 2500 fs. Therefore, the lifetime of the excited state can be regarded as 2400 fs. The experimental results of femtosecond transient spectra [23] show that the excited state lifetime is 6 ps. The calculated lifetime and result experimentally are of the same order of magnitude. Considering the fact that a single TATB molecule was simulated in vacuo without any solvent effect included, it is fair to say the simulation results are consistent with experimental observations.

**Figure 3:**
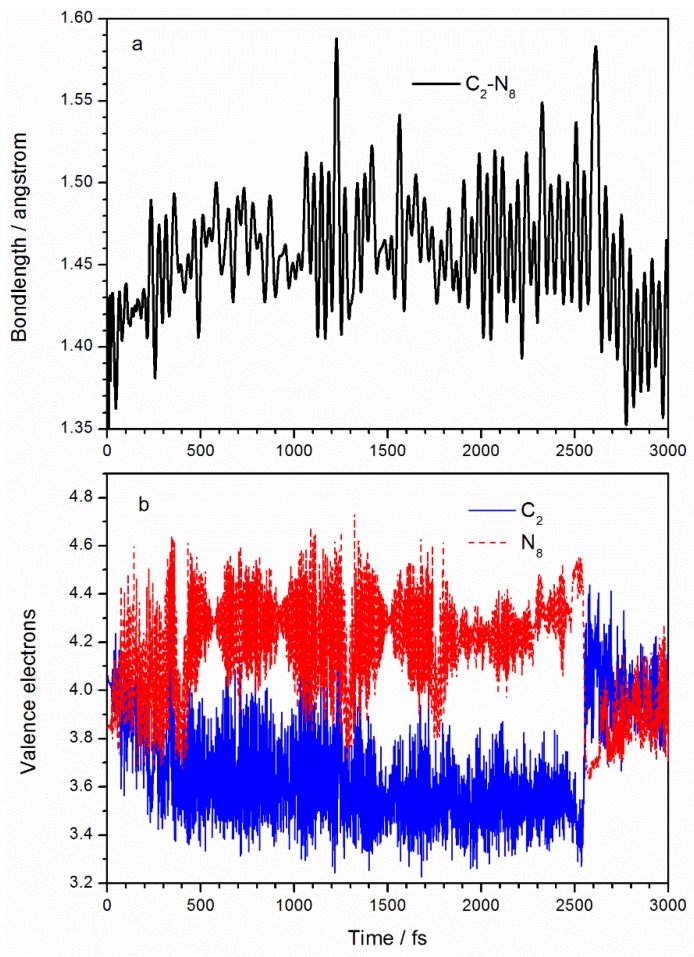
Variations with time of (**a**) C_2_–N_8_ bond length and (**b**) valence electrons of C_2_ and N_8_ atoms in the activated nitro group.

Figure 3a shows how the C_2_–N_8_ bond length changes with time. The average bond length of C_2_–N_8_ in the ground state is 1.40 Å, compared with the optimized ground state molecular structure of 1.431 Å at the MP2/6-311++g(2d,2p) level and 1.438 Å as reported in Reference [23]. After laser irradiation, the bond length of C_2_–N_8_ extends to an average of 1.46 Å. TD-DFT calculations [23] of TATB excited states show that C_2_–N_8_ increases to 1.474 Å in the S_1_ state. After 2500 fs, the C_2_–N_8_ bond returns to its initial value due to the molecular decay to the ground state. In a SERID simulation, the number of electrons at every atom over time is obtained by projecting the electron wavefunction into the atomic orbital. Initially (at 0 fs), the valence electron numbers at C_2_ and N_8_ are 4.1 and 3.8 Å due to O (in the ANG) being highly electronegative. After excitation, it is clear from Figure 3b that the number of C_2_ valence electrons decreases to an average of 3.6 and the number of N_8_ atom valence electrons increases to an average of 4.3, implying that about 0.5 electrons are transferred from the C_2_ to the N_8_ atom. Chu et al. [23] analyzed the charge distributions of the ground state and the first excited state of TATB molecule and found net 0.339-electron from the benzene ring to the ANG due to excitation. According to the frontier orbital properties in Figure 2, the transfer of electrons from C_2_ to N_8_ implies that the number of orbital electrons increases at the C–NO_2_ site, resulting in a decreased bond order, which explains the increased C_2_–N_8_ bond length.

**Figure 4:**
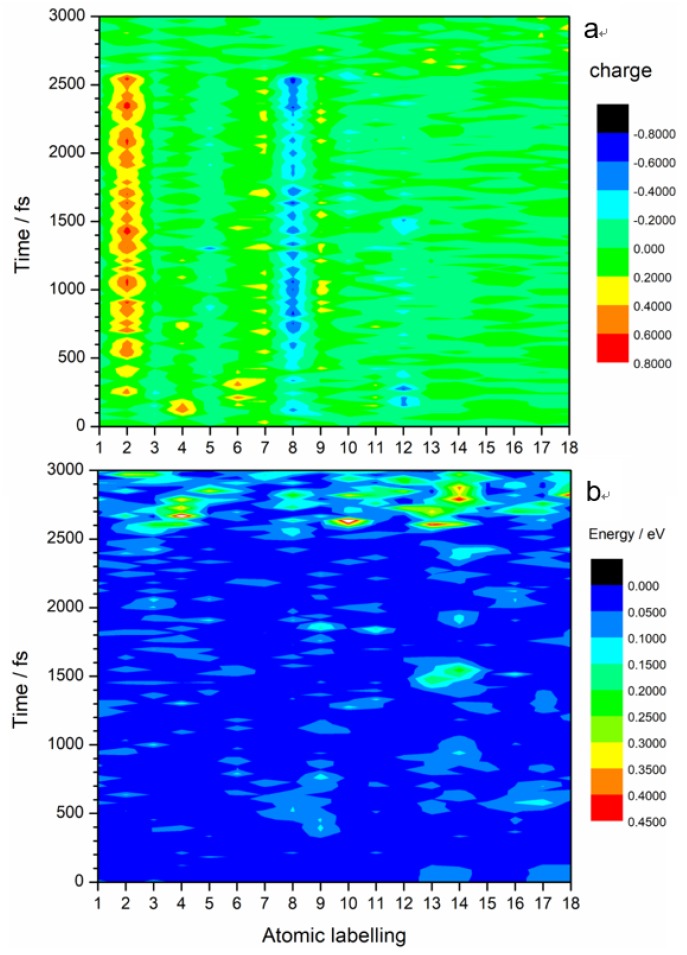
Distribution contours of (**a**) charge and (**b**) kinetic energy of each atom of TATB at current excited state. Atomic labeling displayed in the *x*-axis is the same as in Figure 1a. The hydrogen atoms were ignored.

It is worth noting that almost all the excited state intramolecular electron transfers happen between C_2_ and N_8_ atoms, as shown in Figure 4a. The color map clearly shows charge transfers between N_8_ (light blue–dark blue color) and C_2_ (yellow-red color). Slight charge transfers are observed at N_7_ and N_9_. The kinetic energy distribution diagram (Figure 4b) shows a significant rise in the kinetic energy of O_13_ and O_14_ at 1500 fs. These O atoms are part of the ANG. This suggests that electronic excitation energy is converted to vibrational energy that is mostly concentrated on the ANG. This could possibly lead to reactions that involve C–NO_2_ bond breaking, N–O bond cleavage, and ring opening. Indeed, the breaking away of O atom(s) from nitro is a precursor of the generally accepted NO_2_–ONO isomerization mechanism [16,17]_._ Nitro elimination and formation of fragments by aromatic ring opening observed in other energetic molecules could also happen in TATB.

**Figure 5:**
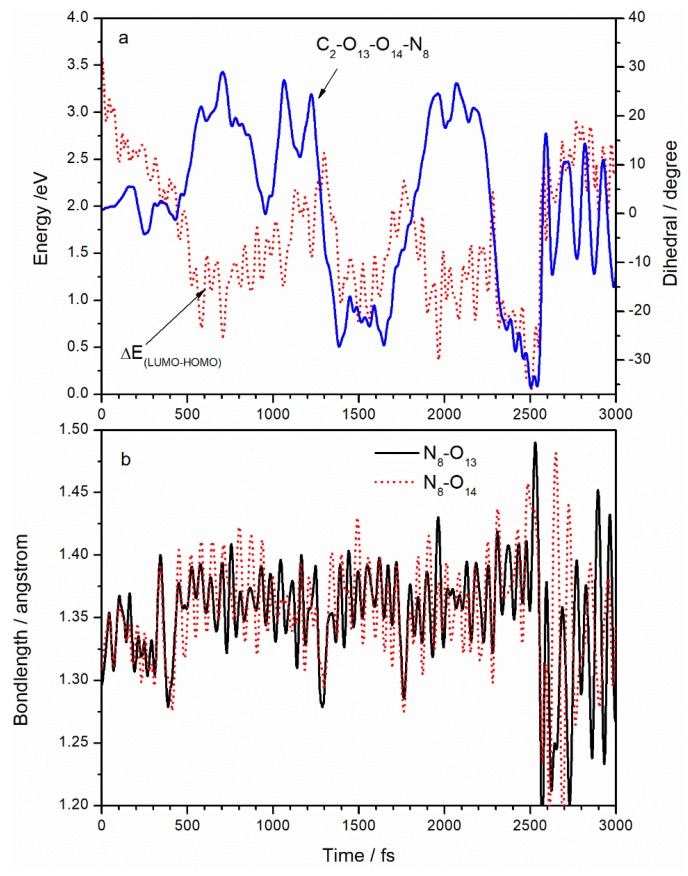
(**a**) Comparison between the energy gap of LUMO–HOMO and N_8_ atom pyramidalization; (**b**) variations with time of N_8_–O_13_ and N_8_–O_14_ bond lengths.

In order to investigate how vibrations affect deactivation, we compared the changes in all the degrees of freedom with the excitation energy. We found that the pyramidalization of the N_8_ atom in the ANG plays an important role is the deactivation of the TATB molecule. Figure 5a displays the variation of the C_2_–O_13_–O_14_–N_8_ dihedral angle and LUMO–HOMO energy gap with time. Initially, with energy gap being 3.2 eV, the C_2_–O_13_–O_14_–N_8_ dihedral angle is about 0°, indicating that the ANG is in the TATB molecular plane. After excitation, the energy gap decreases gradually, accompanied by an increase in the C_2_–O_13_–O_14_–N_8_ dihedral vibration, leading to the pyramidal structure shown in Figure 1c. With the concurrent decrease in the LUMO–HOMO energy gap, we can surmise that the pyramidalization of the C_2_–O_13_–O_14_–N_8_ dihedral angle is the essential vibrational mode associated with the nonradiative deactivation channel. At 2500 fs, the LUMO–HOMO energy gap reaches a minimum of 0.096 eV and the dihedral angle C_2_–O_13_–O_14_–N_8_ reaches a minimum of −35.2°. The N_8_ atom deviates from the planarity to the maximum extent at that time. After deactivation, the ANG still retains a large vibrational amplitude, which suggests the conversion of excited electronic energy to vibrational energy. However, the C_2_–O_13_–O_14_–N_8_ angle has an average value of 0°, suggesting a return to the planar structure. The pyramidal structure has also been found in the (S_1_/S_0_) _CI_ structure of non-adiabatic decay [25,26,27,28] of other small energetic molecules, such as NB, TNA, TNT, and DMNA. Figure 5b displays the variation of N_8_–O_13_ and N_8_–O_14_ distances with time. Starting at 1.296Å, both N_8_–O_13_ and N_8_–O_14_ bonds lengthen after laser application; large fluctuations of about 1.37 Å are observed between 300 and 2500 fs. Compared to Figure 3 and Figure 4, it was found that in the C_2_–O_13_–O_14_–N_8_ dihedral angle, N_8_–O_13_, N_8_–O_14_, and C_2_–N_8_ bonds lengths significantly changed from 300 to 2500 fs. This suggests that activation of the ANG involves electron transfer from C_2_ to N_8_ and increased vibrational amplitude in the ANG as intramolecular hydrogen bonds are broken. At 2500 fs, the N_8_–O_13_ bond reaches a maximum of 1.442 A. N_8_–O_13_ and N_8_–O_14_ bonds sharply vibrate when electrons decay but still have an average value of 1.3 Å, suggesting a return to ground state values. The increased vibrational amplitudes cause the N–O bonds in the ANG to lengthen to as much as 1.5 Å, suggesting a propensity for N–O bond breaking during deactivation.

**Figure 6:**
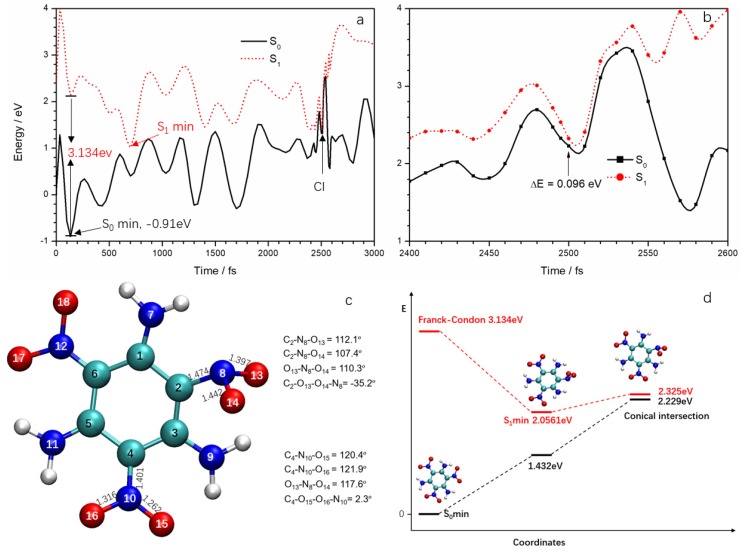
(**a**) S_0_ and S_1_ potential energy surface along with simulation trajectory from TD-B3LYP/cc-pVDZ calculation; (**b**) the detailed variations of S_0_ and S_1_ potential energy surface near the deactivation point (relative to the S_0_ minimum (−0.91 eV) shown in (**a**); (**c**) the structure at the conical intersection (CI); (**d**) the minimum energy pathway from the Franck–Condon (FC) point to the CI.

Since the Ehrenfest dynamics method ignores electron spin, the SERID method cannot identify the multiplicity of excited states in the simulation. The CASSCF method can optimize the structure of different electronic states and the conical intersection of the adiabatic potential energy surface, but it is difficult to calculate for TATB if one were to include all the π electrons and orbitals. Even if some electrons and orbitals are neglected to reduce the active space, it would be hard to choose which ones to neglect because of the lack of the conical intersection geometry on the potential energy surface. Although the simulation found that the excited state is basically located in the ANG, the degrees of freedom (including pyramidalization of N atom, bending of O–N–O, stretch of N–O, and rotation of nitro, etc.) were still too complex for confirming reaction coordinates. For this study, the ground state and the lowest singlet excited state potential energy surfaces were calculated along the simulation trajectory at the unrestricted TD-B3LYP/cc-pVDZ level to explore a sample strategy to investigate deactivation pathways. The potential energy curves of S_0_ and S_1_ are displayed in Figure 6a. We are interested in the deactivation channels, so only the energies of trajectory points between 2400 and 2600 fs are given in Figure 6b. In the potential energy calculation, the ground state energy of the first conformation (i.e., conformation at 0 fs) was assumed to be zero. However, we found the S_0_ curve reached a minimum of −0.91 eV at 135 fs due to conformation and energy fluctuations in the dynamics simulation. Thus, the geometry at 135 fs should be regarded as S_0_ minimum. Therefore, both S_0_ and S_1_ potential curves should be shifted 0.91 eV upward based on this geometry. The S_1_ minimum of 1.146 eV at 700 fs should be increased to 2.056 eV. All the energy values of trajectory points are increased by 0.91 eV in Figure 6b. It can be seen from Figure 6b that at 2500 fs, the energy gap between S_1_ and S_0_ is at a minimum (0.096 eV); this can be regarded as corresponding to a CI. It also means that the configuration at 2500 fs is very close to the structure of TATB at the CI. Therefore, the decay in the trajectory can be regarded as the S_1_→S_0_ non-adiabatic deactivation via CI.

The CI structure is given in Figure 6c. Some critical geometrical parameters are also included. There are significant differences of structure between the ANG and the meta nitro group. In the ground state TATB molecule, the three nitro groups are coplanar with the benzene ring because of the formation of a p–π conjugated system. At the CI, however, the N_8_ atom is off the plane, forming a pyramid with the C_2_, O_13_, and O_14_ atoms and the N at the meta-nitro group remained in plane. At CI, the bond lengths of C_2_–N_8_, N_8_–O_13_, and N_8_–O_14_ were 1.474, 1.397, and 1.442 Å, respectively; these are essentially sigma bonds. The bond angles C_2_–N_8_–O_13_, C_2_–N_8_–O_14_, and O_13_–N_8_–O_14_ were 112.1, 107.4, and 110.3°, respectively. They are very similar to the bond angles in nonequivalent sp^3^ hybridization, as in the NH_3_ molecule. On the other hand, the bond lengths in meta-nitro are shorter than the ones in the ANG and could be regard as π bonds. The bond angles of the meta-nitro are close to 120°, suggesting sp^2^ hybridization for the N atom. In order to determine the relaxation path involved in the fast internal conversion of TATB from the S_1_ state, the minimum energy connecting the Franck–Condon (FC) points of S_1_ and S_0_ surfaces of TATB to the minimum energy (S_1_/S_0_) _CI_ was determined, as seen in Figure 6d. The TD-DFT-calculated vertical excitation energy (here we defined the vertical excitation energy as the S_1_–S_0_ energy gap at S_0_ minimum) for the lowest lying excited electronic states (S_1_) of TATB is 3.134 eV. The calculated vertical excitation energy is in good agreement with the available experimental [24] excitation wavelength of 400 nm (3.1 eV). Direct comparison of the excitation energy used in experimental work with the calculated vertical excitation energy reveals that TATB is excited below the Franck–Condon (FC) point of the S_1_ state at 400 nm. Our calculated results are in close agreement with Xiong et al. [23] at the TD-B3LYP/6-311++G (d, p) level. They found that the vertical excitation energy and one S_1_ minimum were 350.5 kJ·mol^−1^ (3.63 eV) and 218 kJ·mol^−1^ (2.26 eV), respectively.

## 3. Materials and Methods

In this study, the SERID model was used to carry out the dynamics simulation of TATB followed by laser excitation. In the simulation, the electronic motion was calculated by quantum mechanics, while the nuclear motion and radiation field were treated using the classical method. The one-electron states were obtained at each time step by solving the time-dependent Schrödinger equation in a nonorthogonal basis,

(1) $$i\hbar \frac{\partial \Psi_{j}}{\partial t}=S^{-1}\cdot H\cdot \Psi_{j}$$

where **Ψ** is the electronic wavefunction, **S** is the overlap matrix for the atomic orbitals, **H** is Hamiltonian operator. The electronic motion was calculated using the density functional-based tight binding (DFTB) method. The laser pulse was characterized by a vector potential **A**, which is coupled to the Hamiltonian via time-dependent Peierls substitution.

(2) $$H_{ab}\left(X-X\prime \right)=H_{ab}^{0}\left(X-X\prime \right)\exp\left(\frac{iq}{\hbar c}A\cdot \left(X-X\prime \right)\right)$$

Here, $H_{ab}\left(X-X\prime \right)$ is the Hamiltonian matrix element for basis functions *a* and *b* on atoms at X and X′, respectively, and q=−e is the charge of the electron.

The vector potential **A** comprehensively describes the laser pulse (pulse width, photon frequency, pulse intensity).

(3) $$A=A(x,t)=A_{0}\cos\left(\frac{\pi (t-\frac{t_{0}}{2})}{t_{0}}\right)\cos(\omega t)$$

Here, t_0_ is width of pulse, *ω* is angular frequency, *A*_0_ is a constant.

In this model, the nuclear motion is solved by the Ehrenfest equation of motion,

(4) $$M_{l}\frac{d^{2}X_{l\alpha }}{dt^{2}}=-\frac{1}{2}\sum_{j}\Psi_{j}^{+}\cdot \left(\frac{\partial H}{\partial X_{l\alpha }}-i\hbar \frac{1}{2}\frac{\partial S}{\partial X_{l\alpha }}\cdot \frac{\partial }{\partial t}\right)\cdot \Psi_{j}-\partial U_{rep}/\partial X_{l\alpha }$$

where $U_{rep}$ is the effective nuclear–nuclear repulsive potential and $X_{l\alpha }=\langle {\overset{\wedge }{X}}_{la}\rangle$ is the expectation value of the time-dependent Heisenberg operator for the *α* coordinate of the nucleus labeled by *l* (with *α* = *x*, *y*, *z*). Equation (4) is obtained by neglecting the terms of second and higher order in the quantum fluctuations $\hat{X}-\langle {\hat{X}}_{l\alpha }\rangle$ in the exact Ehrenfest theorem.

The Hamiltonian matrix, overlap matrix, and ion–ion repulsive potential are determined in a density-functional-based tight binding (DFTB) method [33,34], which is derived from local density approximation (LDA) functional and does have essentially the same strengths and limitations as TD-DFT. In particular, the bonding is well described but the excited state energies are typically too low. For this reason, as is conventional, we matched the effective central photon energy of the laser pulse to the relevant density-functional (rather than experimental) excitation energy; this should not have a significant effect on the interpretation of the results.

The basis functions used in the present simulations are the 1s atomic orbital of H and the valence s and p orbitals of C. (Spin-up and spin-down states are not distinguished.) The Hamiltonian matrix elements and overlap matrix elements are calculated for a dimer for interatomic distances on a relevant scale and the results from the calculations can then be tabulated and employed in time-dependent simulations.

The weakness of the Ehrenfest method is that it amounts to averaging over all the terms in the Born–Oppenheimer expansion,

(5) $$\Psi^{total}\left(X_{n},x_{e},t\right)=\sum_{i}\Psi_{i}^{n}\left(X_{n},t\right)\Psi_{i}^{e}\left(x_{e},X_{n}\right)$$

rather than following the time evolution of a single term—i.e., a single potential energy surface—which is approximately decoupled from all the others. (Here, $X_{n}$ and $x_{e}$ represent the sets of nuclear and electronic coordinates, respectively, and the $\Psi_{i}^{e}$ values are eigenstates of the electronic Hamiltonian at fixed $X_{n}$.) The strengths of the present approach include the retention of all of the 3N nuclear degrees of freedom (instead of only the 2 or 3 that are typically considered in a potential energy surface calculation) and the incorporation of both the excitation due to a laser pulse and the subsequent de-excitation at an avoided crossing near a conical intersection.

Because the SERID model is based on a mean field theory, it is not clear which electron is excited to a specific electronic excited state. Therefore, it is necessary to assume a reasonable quantum chemistry model. In this study, a TD-DFT method was used to calculate the energy of ground state and excited state every 100 fs in a simulated trajectory (at 10 fs intervals near the deactivation point) to obtain the potential energy surface. This calculation helps to elucidate the non-adiabatic transition of TATB molecules.

The Gaussian 09 quantum chemical package (Gaussian, Inc., Wallingford, CT, 2009) was used for geometry optimizations and TD-DFT calculations. The VMD [35] open source program was used to visualize the molecular structure. 

## 4. Conclusions

In this paper, a photophysical deactivation pathway of laser-excited TATB molecules is reported. The vibration and pyramidalization of the ANG is the key factor of deactivation. Electron transfer from the benzene ring to the ANG was observed. The charge transfer leads to the obvious increase of the C–NO_2_ bond and formation of the C–N σ bond. It is the reason why the ANG can rotate freely in the excited state. The energies of the S_0_ state and the S_1_ excited state of each configuration in the trajectory were calculated by the TD-DFT method. It was found that the energy gap of the nonradiative deactivation in the simulation was 0.096 eV, close to the conical intersection. The vertical excitation energy and S_1_ minimum were also close to experimental values and other theoretical results. The S_0_–S_1_ excitation is localized at the ANG, and the electronic excitation energy is converted to vibrational energy that is mostly concentrated on the ANG. Drastic vibrations of C–NO_2_ and N–O bonds in the ANG suggest that nitro elimination and N–O bond cleavage could happen in TATB subjected to laser irradiation.
